# AlCl_3_ exposure induces nephrotoxicity in mice by mediating ferroptosis through the NRF2 signaling pathway

**DOI:** 10.3389/ftox.2025.1692410

**Published:** 2025-11-26

**Authors:** Liu-Dan Liang, Xiao-Yue Zhao, Li Li, Sheng Liang, Jin-Min Zhang, Jian-Nan Lv, Feng-Lian Deng, Chun-Lei Lu, Qian Li, Qi-Wen Huang, Mei-Jin Huang, Hui-Xin Peng

**Affiliations:** 1 Affiliated Hospital of Youjiang Medical University for Nationalities, Baise, Guangxi, China; 2 Department of Infectious Diseases, Affiliated Hospital of Youjiang Medical University for Nationalities, Baise, Guangxi, China; 3 Guangxi Clinnical Medical Research Center for Hepatobiliary Diseases, Baise, Guangxi, China; 4 Department of Nephrology, Baise People’s Hospital, Baise, Guangxi, China; 5 The Second People’s Hospital of Jinzhong, Jinzhong, Shanxi, China

**Keywords:** ferroptosis, aluminum, ferrostatin-1, nephrotoxicity, biological toxicity

## Abstract

Aluminum is toxic to both humans and animals. Exposure to AlCl_3_ can lead to kidney function damage, yet the specific underlying mechanism remains elusive. This study aimed to investigate whether ferroptosis is involved in the renal toxicity induced by AlCl_3_ exposure in mice and to elucidate its potential molecular mechanism. Forty-eight C57BL mice were randomly assigned to six groups, with eight mice in each group: a control group, low -, medium -, and high - dose aluminum exposure groups, a ferroptosis inhibitor group, and a ferroptosis inhibitor + high - dose aluminum exposure group. Mice in the aluminum exposure groups received intraperitoneal injections of different doses of AlCl_3_ solution for 4 weeks (5 times per week), while the ferroptosis inhibitor group was intraperitoneally injected with Fer - 1 for 4 weeks (2 times per week). After the experimental period, multiple indicators were examined. The results demonstrated that AlCl_3_ exposure impaired the renal function and structure of mice. It also led to an increase in lipid peroxidation products, Fe^2+^, and Al content in renal tissue. Moreover, the expression levels of genes and proteins such as GPX4 and Nrf2 were decreased, whereas the expression levels of the ACSL4 gene and protein were increased.However, after pretreatment with Fer - 1, the aforementioned indicators were ameliorated. Specifically, the expression of ACSL4 decreased, and the expression of GPX4 and other related factors increased.In conclusion, this study suggests that AlCl_3_ exposure may trigger ferroptosis in renal tissue cells by inhibiting the NRF2 pathway, thereby causing kidney function damage in mice. These findings provide a novel perspective on the mechanism of AlCl_3_ - induced renal toxicity.

## 1 Introduction

The presence of aluminum (Al), a highly abundant metallic element on earth, has garnered considerable attention due to its biotoxicity. Al serves various purposes, including its use as a food additive, in cookware with an Al content of approximately 20%, as a component in drinking water at a concentration of 0.2 mg/L, in water purification systems, canning jars, Al foil, and antiperspirant cosmetics. Although the gastrointestinal tract has a limited capacity to absorb Al (less than 1%), the metal can accumulate in vital organs such as the kidney, liver, and brain over time, resulting in considerable cytotoxicity (Othman et al., 2020). The mechanism of Al toxicity has emerged as a significant research focus in recent years, particularly regarding its nephrotoxic effects. Al can induce histopathological alterations in the kidney, including glomerular atrophy, tubular necrosis, mitochondrial degeneration, and vacuolization of organelles (El-Kenawy Ael et al., 2014). These processes ultimately impact the glomerular filtration and tubular reabsorption rates, resulting in renal insufficiency (Ghorbel et al., 2016). Investigations into the mechanisms of kidney injury have substantiated that Al can trigger oxidative stress (Liu et al., 2016), DNA damage (Jalili et al., 2020), mitochondrial damage, apoptosis (Liu et al., 2022), fibrosis (Wei et al., 2023a), and alterations in cell membrane composition (Kriegel et al., 2020). In addition, AlCl_3_ activates and stimulates the release of inflammatory mediators from renal cells, inducing an inflammatory response and accelerating renal tubular cell death (Ahmed et al., 2022). Research on the molecular mechanisms associated with Al-induced nephrotoxicity remains limited. Therefore, the objective of the present investigation was to evaluate the molecular mechanisms underlying AlCl_3_-induced nephrotoxicity, offering a fresh perspective on mitigating AlCl_3_-induced renal injury.

Ferroptosis is a newly identified type of regulated cell death characterized by iron ion accumulation, cellular redox metabolism disruption, and excessive accumulation of lipid peroxides and reactive oxygen species (ROS) (Bayır et al., 2020; Yang and Stockwell, 2016). Excessive Al competes with iron for binding to ferritin and transferrin, thereby impacting iron transport. Additionally, Al can displace Fe^2+^ from cellular reserve sites, increasing free Fe^2+^ concentration and stimulating iron-mediated free radical production (Lanser et al., 2021). Al promotes neuronal ferroptosis by disrupting iron homeostatic balance and inducing oxidative stress (Aschner et al., 2022). Moreover, recent research conducted by Zhang et al. (2020) and Cheng et al. (2021) has provided further evidence supporting the critical role of ferroptosis in Al-induced neuronal death. These researchers discovered that aluminium can disrupt the redox system in the hippocampus of rats. This disruption leads to a decrease in glutathione (GSH) levels, an increase in reactive oxygen species, the triggering of ferroptosis in hippocampal cells, and ultimately cognitive decline (Zhang et al., 2021). Additionally, Al can induce GSH depletion, inactivate glutathione peroxidase (GPX), cause ROS accumulation, and trigger ferroptosis in PC12 cells (Liang et al., 2025).

Nuclear factor erythroid 2-related factor 2 (Nrf2) regulates dozens of antioxidant genes (Baird and Yamamoto, 2020). In the physiological state, Nrf2 can form a complex with its negative regulator, Kelch-like ECH-associated protein 1 (Keap1), in the cytoplasm. Subsequently, Keap1 targets Nrf2 for ubiquitination and proteasomal degradation, maintaining Nrf2 signaling at low levels. However, when exposed to stimuli like oxidative stress or electrophilicity, Nrf2 forms a weak bond with Keap1, resulting in decreased ubiquitination and degradation of Nrf2. When Nrf2 is enriched, its protein level rises, resulting in its translocation into the nucleus. There, it interacts with antioxidant response elements in the promoter regions of target genes, thereby promoting the transcription of its downstream genes, including HO1, NQO1, glutathione s-transferase, and γ-glutamylcysteine synthetase. This process facilitates detoxification, antioxidant, and anti-inflammatory effects (Yamamoto et al., 2018). Emerging research has highlighted the involvement of Nrf2 in ferroptosis induced by lipid peroxide accumulation (Cui et al., 2021; Vasileva et al., 2020). The activation of Nrf2, resulting in its dissociation from Keap1 triggers the antioxidant response of the body to eliminate excessive ROS and inhibit ferroptosis (Ren et al., 2021).

Although current research has demonstrated that aluminium (Al) can cause kidney damage, numerous gaps remain in our understanding of the specific mechanisms involving ferroptosis and nuclear factor erythroid 2 - related factor 2 (Nrf2). At the ferroptosis level, while it is recognized that Al exposure can induce oxidative stress and lipid peroxidation in kidney cells, which aligns with the hallmarks of ferroptosis, it remains uncertain whether ferroptosis represents the core pathological process underlying Al - induced kidney damage. Specifically, the precise signaling pathway through which Al triggers ferroptosis in kidney cells is unknown. Moreover, the role and contribution of ferroptosis at different stages of kidney injury (initiation, progression, and deterioration) have yet to be elucidated. Nrf2, a key antioxidant transcription factor in cells, plays a crucial role in maintaining redox homeostasis. However, the specific manner in which Al exposure regulates the activity and expression of Nrf2 in the kidneys is not fully understood.

This study comprehensively explored the mechanisms of ferroptosis and the nuclear factor erythroid 2 - related factor 2 (Nrf2) pathway in the renal toxicity of aluminum chloride (AlCl_3_) in mice. It was revealed that ferroptosis is a critical factor in Al - induced kidney damage. Targeted development of ferroptosis inhibitors or drugs regulating iron metabolism could offer novel therapeutic targets for preventing and treating Al - induced renal toxicity.Simultaneously, the specific role of the Nrf2 pathway was elucidated. By activating Nrf2 or modulating the expression of its downstream antioxidant genes, the antioxidant capacity of kidney cells can be enhanced, effectively mitigating the oxidative stress and kidney damage induced by Al. These findings provide a foundation for further preventive and therapeutic strategies against AlCl_3_-induced kidney injury.

## 2 Materials and methods

### 2.1 Reagents and materials

Aluminum trichloride hexahydrate (AlCl_3_-6H_2_O) was procured from Shanghai Aladdin Biochemical Science and Technology Co Ltd (L1706080). Electro SDS-PAGE rapid electrophoresis solution (G4839), electrotransfer solution (D1060), serum-free rapid blocking solution (SW3012), and hematoxylin-eosin staining kit (G1120) were procured from Solarbio. BCA kit (ZJ101L) and PSMF-containing protein lysate (PC101) were obtained from Shanghai Yazyme Biotechnology Co., Ltd. Malondialdehyde (MDA) assay kit (S0131M), superoxide dismutase (SOD) kit (S0101M), and glutathione (GSH) kit (S0053) were sourced from Beyotime Biotechnology, China. ROS staining solution (D7008) was obtained from Sigma, U.S.A. 4′,6-diamidino-2-phenylindole dihydrochloride (DAPI) staining solution (E-CK-A163) was procured from Wuhan Elabscience. The tissue iron assay kit (A039-2-1) was sourced from Nanjing Jianjian Bioengineering Institute, China. Ferrostatin-1 (347174-05-4) was obtained from MCE, China. Nrf2 antibody (80593-1-RR) was procured from Wuhan Sanying Biology Technology Co., Ltd, China. HO-1 antibody (R24541), Glutathione peroxidase 4(GPX-4) antibody (381,958), Ferritin heavy chain(FTH) antibody (R23306), and Solute Carrier Family 7 Member 11(SLC7A11) (382036) were sourced from Zhengneng, China. Acyl - CoA synthetase long - chain family member 4(ACSL4) (ab155282) was obtained from Abcam, United Kingdom. A horseradish peroxidase-labeled secondary antibody (#S0001) was procured from Affinity, China. SYBR Green RT-qPCR kit (22,204) was sourced from TOLOBIO, China.

### 2.2 Animals and grouping

Forty-eight male mice, aged 4 weeks and weighing 18–20 g, were obtained from Changsha Tianqin Biotechnology Co., Ltd. They were housed in a controlled environment at 22 °C–25 °C and 50%–60% humidity. All mice were provided with standard feed and maintained in a pathogen-free environment. The animal feeding and treatment protocols were reviewed and approved by the Welfare and Ethics Review Committee of Changsha Tianqin Biotechnology Co., Ltd. All procedures were conducted per the regulations and guidelines set forth by the relevant institutions and government authorities regarding animal ethics. Based on the chronic oral minimal risk level (MRL) of 1 mg Al/kg·day for healthy individuals recommended by the Agency for Toxic Substances and Disease Registry (ATSDR) of the United States Centers for Disease Control and Prevention, a scaling estimation method based on the no - observed - adverse - effect level (NOAEL) was employed. The calculation formula is as follows: the mouse dose = 1 mg/kg·day × 1/0.0811 ≈ 12.35 mg/kg·day, where 0.0811 is a fixed conversion coefficient. This approach was combined with the aluminum exposure model established by Wei et al. (2023b). Interventions were conducted via intraperitoneal injection in the following groups: 1. Control group (Group C, receiving 0 mg/kg/d of AlCl_3_). 2. Low - dose aluminum exposure group (Group L, receiving 5 mg/kg/d of AlCl_3_). 3. Medium - dose aluminum exposure group (Group M, receiving 10 mg/kg/d of AlCl_3_). 4. High - dose aluminum exposure group (Group H, receiving 20 mg/kg/d of AlCl_3_). 5. Ferroptosis inhibitor group (Group F, receiving 5 mg/kg/d of Fer - 1). 6. High - dose aluminum exposure + ferroptosis inhibitor group (Group HF, receiving 20 mg/kg/d of AlCl_3_and 5 mg/kg/d of Fer - 1) (Cheraghi et al., 2017; Li et al., 2012). When administering the two reagents simultaneously, AlCl_3_ was injected first, and 3 h later, Fer - 1 was injected into the opposite side of the abdominal cavity (Han et al., 2022).

The preparation of both the drugs and the aluminium solution was carried out using normal saline. Following the pharmacological intervention, mice were euthanized by administering 200 mg/kg of sodium pentobarbital. Subsequently, intraperitoneal venous blood was collected from the mice, and the kidney tissues were quickly collected on ice. Half of the kidney tissues were fixed with 4% paraformaldehyde for subsequent HE and immunofluorescence (IF) staining. The remaining half of the kidney tissue was stored at −80 °C in the refrigerator for protein and RNA assays.

### 2.3 Evaluation of kidney function employing automatic biochemical analyzer

Initially, 1.5 mL of blood was extracted from the abdominal vein of mice and allowed to sit for 30 min. Afterward, it was centrifuged at 3,500 r-min^-1^ with a centrifugation radius of 16 cm. Following centrifugation, the serum was isolated and the serum levels of Scr and Bun were determined by an automatic biochemical analyzer (AU680, Beckman Coulter, Inc. United States).

### 2.4 Determination of aluminum content in mouse kidney tissues by ICP-AES

Mouse kidney tissue was collected and transferred to a container. Subsequently, 5.0 mL of nitric acid was introduced to the container, and the mixture was dried at low temperatures. Then, 1.0 mL of perchloric acid was added to the container until white smoke appeared, forming a colorless solution. The 10% (v/v) hydrochloric acid solution was transferred to a 10 mL fixed-volume measuring flask. Three blank tests were carried out during sample processing. The Al content was determined utilizing inductively coupled plasma-atomic emission spectrometry (ICP-AES) under specified operating conditions.

### 2.5 Detection of Fe^2+^, MDA, SOD, and GSH levels in kidney tissue

Mouse kidney tissues were lysed with RIPA for 30 min and then ground on ice with a glass homogenizer. Afterward, they were washed thrice with pre-cooled PBS to prepare a 10% tissue homogenate. Subsequently, the samples were centrifuged at 12,000 r-min^-1^ for 15 min with a centrifugation radius of 13 cm to extract total protein from the supernatant. The concentration of extracted renal tissue proteins in each mouse group was determined using a BCA kit. Fe^2+^ levels were assessed colorimetrically at 520 nm utilizing a microplate reader. Meanwhile, MDA levels were determined using the TBA method at 532 nm, employing a microplate reader. Additionally, SOD and GSH levels were determined at 450 nm and 410 nm using the microplate reader, strictly following the requirements of the kit.

### 2.6 Observation of rat kidney histopathological morphology utilizing hematoxylin-eosin (HE) staining

The mouse kidney tissue was immersed in a 10% formaldehyde buffer for 48 h, followed by routine dehydration, transparency treatment, and embedding. Subsequently, 5 μm wax sections were deparaffinized utilizing xylene, hydrated with gradient ethanol, stained with hematoxylin for 3 min, and rinsed with distilled water. The samples were differentiated with 1% hydrochloric acid alcohol for a few seconds and then rinsed with running water. They were reblued with 0.5% ammonia, followed by another rinse with running water. Subsequently, the samples were stained with eosin for 1 min and made transparent using gradient ethanol and xylene. Finally, resin-sealed slices were evaluated under an optical microscope to observe any morphological changes in renal tissue.

### 2.7 Detection of ROS levels in kidney tissues via immunofluorescence

The mouse kidney tissues were rapidly immersed in liquid nitrogen and transferred to a −80 °C refrigerator. Subsequently, the kidney tissues were cryosectioned into 3 μm thick slices and incubated with drops of ROS staining solution for 30 min. Subsequently, any excess liquid on the tissue surfaces was carefully removed. After restaining the nuclei with DAPI, the sections were incubated for 10 min and sealed with glycerol. Finally, the sections were evaluated under a fluorescence microscope, and images were captured.

### 2.8 Detection of Nrf2 expression in kidney tissues via immunofluorescence

The frozen mouse kidney tissue sections were thawed at room temperature for 10 min. Subsequently, they were hydrated for 30 min and air-dried to remove surface water. Afterward, a 10% sheep serum blocking solution was prepared utilizing PBS and left at room temperature for 60 min. After discarding the blocking solution, the primary antibody was added, and the sections were incubated overnight at 4 °C (Nrf2 concentration is 1:500). The next day, the frozen sections were allowed to warm up for 30 min at room temperature before being washed. Subsequently, the secondary antibody was added and incubated in the dark for 60 min (Concentration of 1:1,000). Following incubation with the secondary antibody, the sections were washed three times with pre-cooled PBS for 5 min each. Following this, a few drops of diluted DAPI staining solution were added to the tissue sections, which were then stained for 10 min. After rinsing away the staining solution with running water, any excess water was removed by blotting with filter paper. Finally, a drop of fluorescent sealing solution was added to the samples, which were examined under a fluorescence microscope and photographed.

### 2.9 RT-qPCR analysis

To evaluate the purity and concentration of sample RNA, total RNA from each group of kidney tissues was extracted individually employing Trizol reagent. Subsequently, the qualified RNA was reverse-transcribed into first-strand cDNA following the instructions provided with the reverse transcription kit. Afterward, mRNA expression levels were measured using SYBR Green RT-qPCR. The obtained cDNA was utilized as a template and GAPDH as an internal reference. The relative expression of GPX4, ACSL4, HO-1, FTH, Nrf2, and SLC7A11 was analyzed using the relative quantification 2^−ΔΔ^Ct method. The primer sequences are detailed in Table 1.

**TABLE 1:** Primer sequences for RT-qPCR.

Gene	Forward primer(5′- 3′)	Reverse primer(5′- 3′)
FTH1	CGA​GAT​GAT​GTG​GCT​CTG​AA	GTG​CAC​ACT​CCA​TTG​CAT​TC
GPX4	ACT​GCA​ACA​GCT​CCG​AGT​TC	CGA​TGT​CCT​TGG​CTG​AGA​AT
HO-1	TCC​TTG​TAC​CAT​ATC​TAC​ACG​G	GAG​ACG​CTT​TAC​ATA​GTG​CTG​T
SLC7A11	GTG​TTC​GCT​GTC​TCC​AGG​TT	CAG​AGG​AGT​GTG​CTT​GTG​GA
NRF2	ATC​AAC​TAC​CCG​TTC​GAG​AAG	ACT​TGG​TCA​TGT​CGA​TGT​CAT​A
GAPDH	CTG​GAG​AAA​CCT​GCC​AAG​TAT​G	GGT​GGA​AGA​ATG​GGA​GTT​GCT

### 2.10 Western blotting (WB) analysis

We weighed an appropriate amount of kidney tissue, added an appropriate volume of RIPA lysis buffer and PMSF protease inhibitor at a ratio of 100:1, and then thoroughly homogenized the mixture using a high - speed tissue grinder. The homogenate was incubated on ice for 30 min and subsequently centrifuged at 4 °C for 15 min to obtain the supernatant, which represented the total tissue protein. The protein concentration was determined using the BCA method, and the protein was adjusted to a concentration of 2 μg/μL. For electrophoresis, the stacking gel was run at 80 V, and the separating gel was run at 120 V until the dye front reached the bottom of the gel, with a total running time of approximately 90 min. The selection of electrophoresis gels was based on the molecular weight of the target proteins: 12.5% gels were used for proteins with a molecular weight below 20 kDa, 10% gels for those with a molecular weight between 20 kDa and 100 kDa, and 7.5% gels for proteins with a molecular weight above 100 kDa. During the transfer process, for proteins with a molecular weight below 70 kDa, the transfer was carried out at a current of 300 mA for 30 min; for those above 70 kDa, the transfer was at 300 mA for 50 min. The membrane was blocked with 2.5% skimmed milk at 25 °C for 1 h. The primary antibodies, all rabbit - derived, were incubated overnight at 4 °C. Then, an HRP - labeled goat anti - rabbit secondary antibody (at a concentration of 1:5,000) was added and incubated at room temperature for 1 h. A hyper - sensitive ECL solution was used for imaging. The results were analyzed using ImageJ image - processing software. The primary antibodies were all rabbit - derived, and their specific concentrations were as follows: Nrf2 at 1:1,500, GPX4 at 1:1,500, ACSL4 at 1:1000, HO - 1 at 1:1500, FTH at 1:2,000, and SLC7A11 at 1:2000.

### 2.11 Statistical analysis

Data were evaluated employing SPSS 20.0 software, while images were analyzed utilizing Graph Prism 8.0 software. Initially, the data were assessed for normality. Data conforming to a normal distribution were then subjected to statistical comparisons. The comparison between groups was conducted using a one-way analysis of variance (ANOVA). Measurement data were expressed as mean (M) ± standard deviation (SD), whereas enumeration data were expressed as constituent ratio (%). A *P*-value of less than 0.05 reflected statistical significance.

## 3 Results

### 3.1 Basic conditions and body weight changes of mice in each group

During the experiment, mice in the control group exhibited normal development, displayed good fur color, and remained active. Those in the low-dose group showed no obvious abnormality. However, mice in the middle- and high-dose groups exhibited decreased responsiveness, slightly reduced activity, and darker fur. No fatalities occurred during the experiment. Moreover, no statistical differences were observed in the body weights of the mice across the groups prior to the intervention. However, mice exposed to AlCl_3_ exhibited significantly lower body weights and body weight change percentages compared to the control group by the end of the experiment. This trend showed a dose-dependent relationship with AlCl_3_ exposure. Similarly, no significant deviations were observed in the body weight or body weight change percentage among mice in the control and Fer-1 groups at the end of the experiment. However, body weight and body weight change percentage were significantly higher in the FH group than in the H group, suggesting Fer-1 mitigated the weight loss induced by AlCl_3_ exposure (Table 2).

**TABLE 2:** Comparison of body weight between groups.

Parameters	C	L	M	H	F	FH
Initial body weight (g)	19.5 ± 0.72	19.49 ± 0.68^*^	19.58 ± 0.70^**^	19.45 ± 0.76^***^	19.54 ± 0.69^#^	19.46 ± 0.65^##^
Significance		P = 0.972	P = 0.831	P = 0.887	P = 0.915	P = 0.972
Final body weight (g)	26.2 ± 0.65	24.23 ± 0.63^*^	23.23 ± 0.64^**^	20.39 ± 0.73^***^	26.14 ± 0.68^#^	23.25 ± 0.47^##^
Significance		P < 0.001	P < 0.001	P < 0.001	P = 0.845	P < 0.001
Body weight gain (g)	6.7 ± 0.55	4.74 ± 0.96^*^	3.65 ± 0.16^**^	0.94 ± 0.23^***^	6.6 ± 0.49^#^	3.79 ± 0.36^##^
Significance		P < 0.001	P < 0.001	P < 0.001	P = 0.705	P < 0.001

^a^ C compared to L; **C compared to M; ***C compared to H; #C compared to F; ##H compared to FH.

### 3.2 Impact of Al levels in mouse kidney tissues across all groups

**FIGURE 1:**
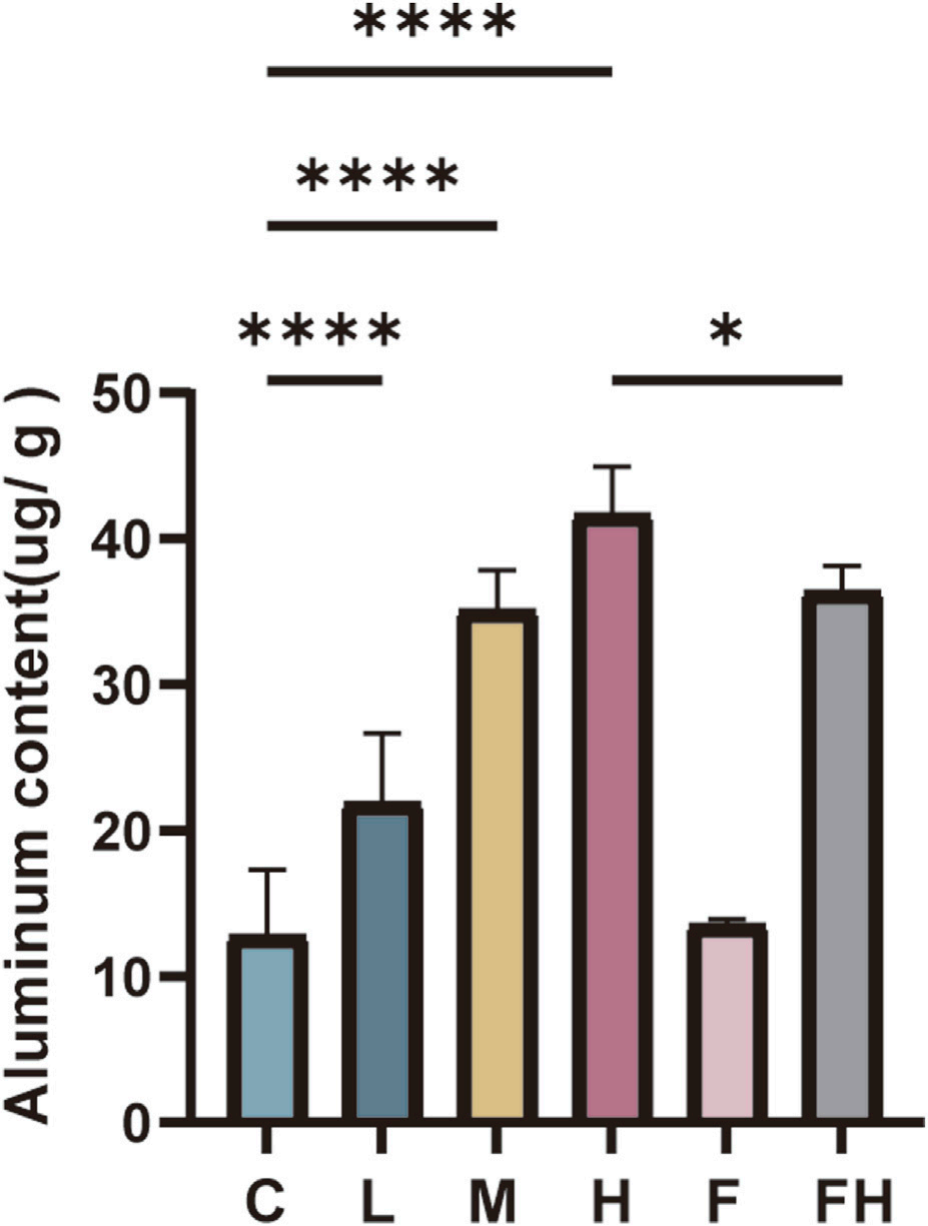
C represents the control group, L represents the low-dose AlCl_3_ exposure group, M represents the medium-dose AlCl_3_ exposure group, H represents the high-dose AlCl_3_ exposure group, F represents the fer inhibitor group, and FH represents the fer + high-dose AlCl_3_ exposure group. (A): AlCl_3_ exposure and Fer intervention changes in aluminum content in kidney tissue after AlCl_3_ exposure. (n = 8, *p < 0.05, **p < 0.01, ***p < 0.001, ****p < 0.0001).

The Al content in the kidney tissues of mice in each group was assessed after 28 days of AlCl_3_ exposure. As the AlCl_3_ exposure concentration increased, the Al content in renal tissues demonstrated an upward trend. Relative to group C, the Al content in kidney tissues of rats in groups L, M, and H were significantly elevated in a dose-dependent manner. Conversely, the Al content in kidney tissues decreased in group FH compared to group H (Figure 1A).

### 3.3 Levels of biochemical parameters related to serum renal function

Creatinine and urea nitrogen levels were assessed in each group of mice to characterize the impacts of AlCl_3_ exposure on renal function. This exposure induced varying degrees of renal impairment in a concentration-dependent manner. Blood creatinine and urea nitrogen levels were significantly higher in mice from groups M and H compared to group C (*P* < 0.05), with the highest levels observed in group H. This indicates that exposure to AlCl_3_ could potentially induce renal injury.

**FIGURE 2:**
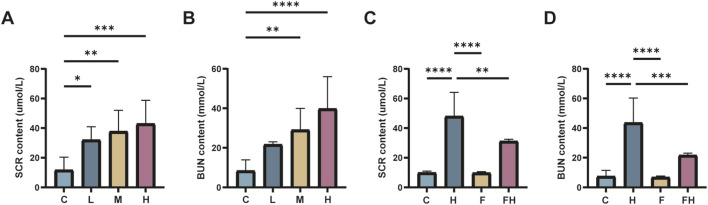
C represents the control group, L represents the low-dose AlCl_3_ exposure group, M represents the medium-dose AlCl_3_ exposure group, H represents the high-dose AlCl_3_ exposure group, F represents the fer inhibitor group, and FH represents the fer + high-dose AlCl_3_ exposure group. **(A)** Changes of serum creatinine in mice exposed to AlCl_3_; **(B)** Changes of urea nitrogen in mice after exposure to AlCl_3_; **(C)** Changes of serum creatinine in mice after fer intervention of AlCl_3_ exposure; D: Changes of urea nitrogen in mice after fer intervention of AlCl_3_ exposure. (n = 8, *p < 0.05, **p < 0.01, ***p < 0.001, ****p < 0.0001).

To further assess whether Fer-1 could reverse AlCl_3_-induced renal impairment, blood creatinine, and urea levels were assessed in the Fer-1 and FH groups. No significant changes were observed in creatinine and urea nitrogen levels in mice administered Fer-1 alone compared to the control group. However, in the FH group, blood creatinine and urea levels were significantly lower than those in the H group, indicating potential involvement of ferroptosis in AlCl_3_-induced renal injury (Figure 2).

### 3.4 Fe^2+^, MDA, SOD, and GSH levels in kidney tissues of mice in each group

**FIGURE 3:**
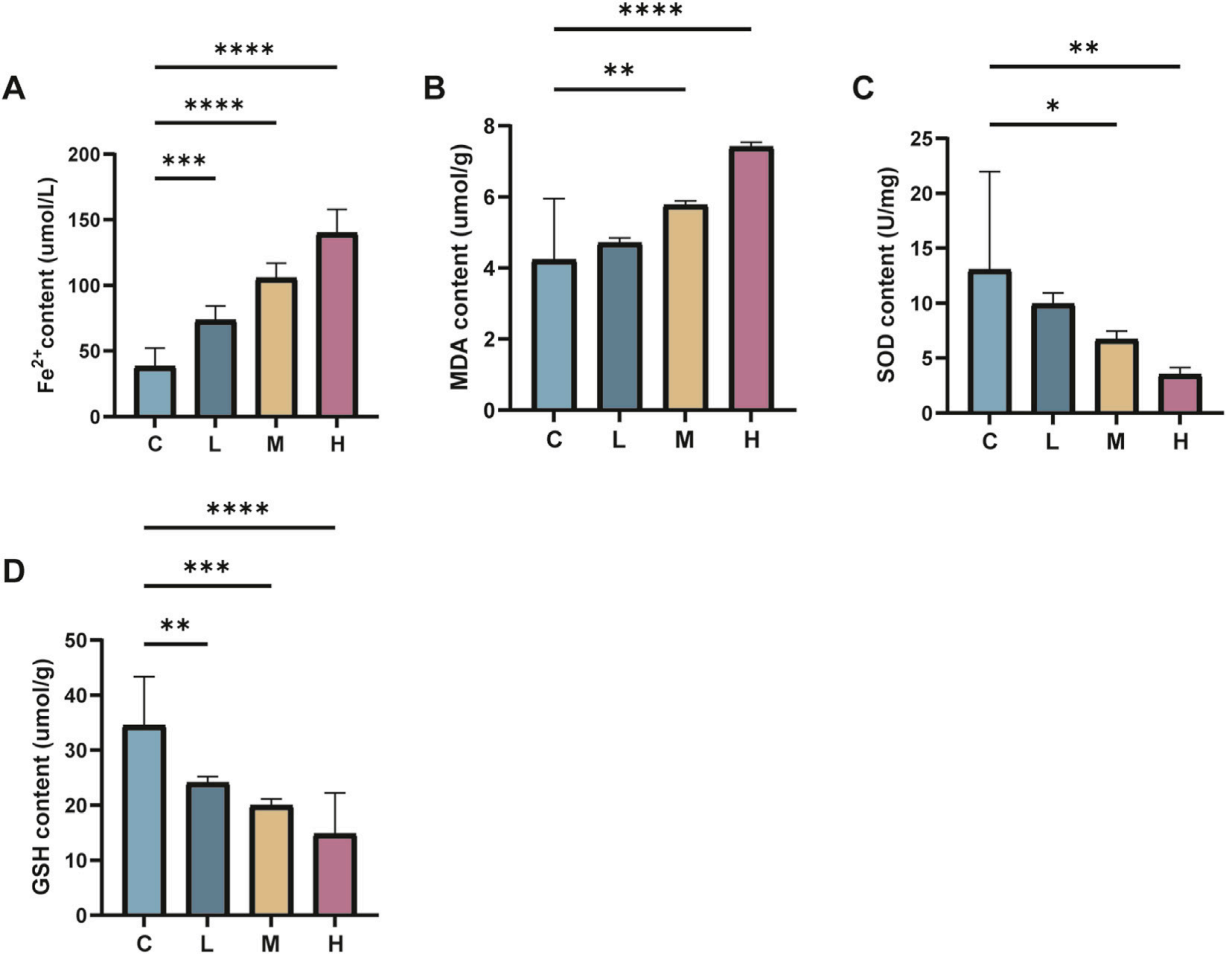
C represents the control group, L represents the low-dose AlCl_3_ exposure group, M represents the medium-dose AlCl_3_ exposure group, H represents the high-dose AlCl_3_ exposure group, F represents the fer inhibitor group, and FH represents the fer + high-dose AlCl_3_ exposure group. **(A–D)** showed the changes of Fe^2+^, MDA, SOD and GSH in renal tissue of mice after exposure to AlCl_3_. E,F,G,H are the changes of Fe^2+^, MDA, SOD and GSH in renal tissue of mice after fer intervention with AlCl_3_ exposure. (n = 8, *p < 0.05, **p < 0.01, ***p < 0.001, ****p < 0.0001).

**FIGURE 4:**
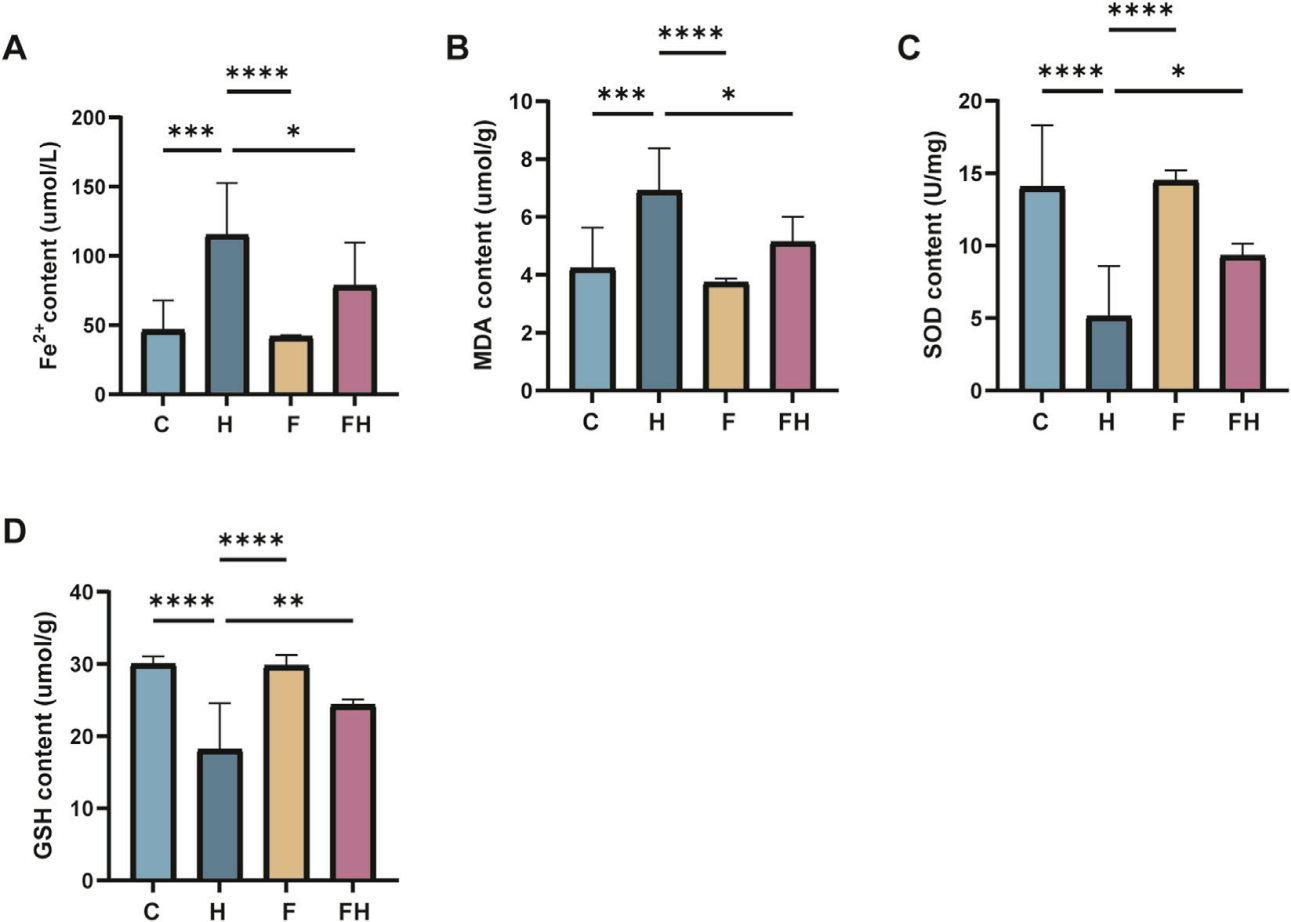
C represents the control group, L represents the low-dose AlCl_3_ exposure group, M represents the medium-dose AlCl_3_ exposure group, H represents the high-dose AlCl_3_ exposure group, F represents the fer inhibitor group, and FH represents the fer + high-dose AlCl_3_ exposure group. **(A–D)** are the changes of Fe^2+^, MDA, SOD and GSH in renal tissue of mice after fer intervention with AlCl3 exposure. (n = 8, *p < 0.05, **p < 0.01, ***p < 0.001, ****p < 0.0001).

To further confirm the induction of iron overload and lipid peroxidation injury in mouse kidney tissues due to AlCl_3_ exposure, levels of Fe^2+^, MDA, GSH, and SOD were assessed. There were no significant differences in Fe^2+^, MDA, GSH, and SOD levels between the Fer-1 group and the control group. However, the AlCl_3_-exposed group exhibited significantly higher ferric ion, Fe^2+^, and MDA levels. Additionally, there were substantially lower levels of GSH and SOD in this group, demonstrating a dose-dependent correlation with AlCl_3_ exposure. Moreover, Fer-1 treatment could reverse the above trends to a certain extent (Figures 3, 4; Tables 3, 4).

**TABLE 3:** Comparison of Fe^2+^ levels and MDA, SOD, and GSH in renal tissues under different concentrations of AlCl3( $\bar{x}$ ± s).

Parameters	control	L	M	H
Fe^2+^(umol/L)	42.01 ± 1.24	81.16 ± 1.46^*^	111.80 ± 1.96^**^	132.48 ± 1.96^***^
Significance		P < 0.001	P < 0.001	P < 0.001
MDA(umol/g)	3.73 ± 0.13	4.71 ± 0.14^*^	5.77 ± 0.11^**^	7.41 ± 0.13^***^
Significance		P < 0.001	P < 0.001	P < 0.001
SOD(U/mg)	14.55 ± 0.75	9.94 ± 0.98^*^	6.73 ± 0.74^**^	3.51 ± 0.63^***^
Significance		P < 0.001	P < 0.001	P < 0.001
GSH(umol/g)	30.01 ± 1.03	24.11 ± 1.10^*^	19.97 ± 1.19^**^	13.02 ± 1.11^***^
Significance		P < 0.001	P < 0.001	P < 0.001

^a^ C compared to L; **C compared to M; ***C compared to H.

**TABLE 4:** Comparison of Fe^2+^ levels, MDA, SOD, and GSH in renal tissues after intervention with ferroptosis inhibitors( $\bar{x}$ ± s).

Parameters	control	H	Fer-1	F + H
Fe^2+^(umol/L)	42.01 ± 1.24	132.48 ± 1.96^#^	41.89 ± 0.99^##^	71.91 ± 1.59^###^
Significance		P < 0.001	P < 0.001	P < 0.001
MDA(umol/g)	3.73 ± 0.13	7.41 ± 0.13^#^	3.74 ± 0.13^##^	5.39 ± 0.12^###^
Significance		P < 0.001	P < 0.001	P < 0.001
SOD(U/mg)	14.55 ± 0.75	3.51 ± 0.63^#^	14.49 ± 0.70^##^	9.31 ± 0.82^###^
Significance		P < 0.001	P < 0.001	P < 0.001
GSH(umol/g)	30.01 ± 1.03	13.02 ± 1.11^#^	29.80 ± 1.47^##^	24.37 ± 0.74^###^
Significance		P < 0.001	P < 0.001	P < 0.001

^a^ C compared to H; ##H compared to Fer - 1; ###H compared to F + H.

### 3.5 Hematoxylin and eosin staining results

**FIGURE 5:**
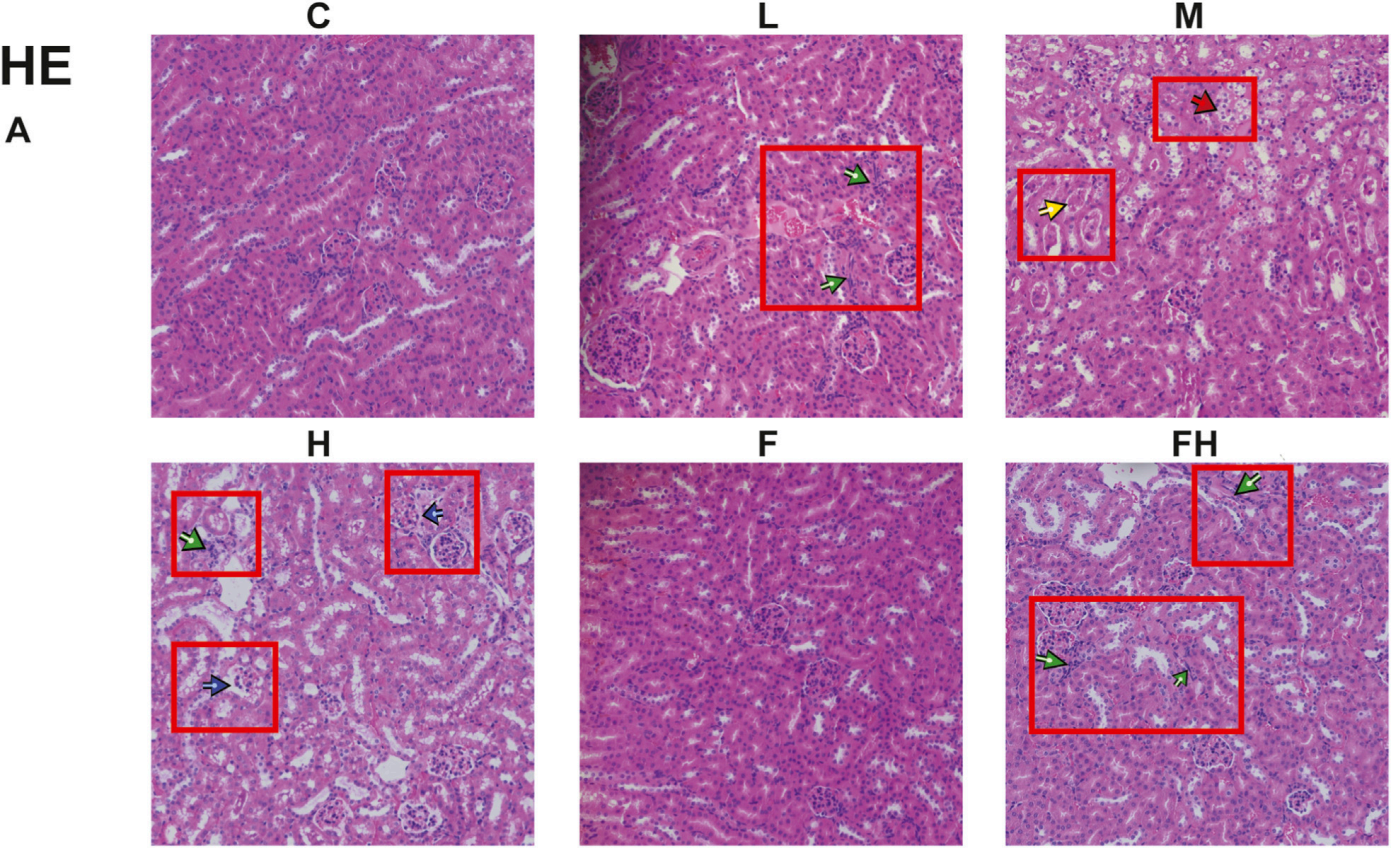
C represents the control group, L represents the low-dose AlCl_3_ exposure group, M represents the medium-dose AlCl_3_ exposure group, H represents the high-dose AlCl_3_ exposure group, F represents the fer inhibitor group, and FH represents the fer + high-dose AlCl_3_ exposure group. **(A)** AlCl_3_ exposure and fer intervention on HE in mice exposed to AlCl_3_. Green arrows indicate inflammatory infiltration; Red represents tubular damage; Yellow represents protein tube type; Blue represents glomerular damage.

The kidneys of control and Fer-1 group mice exhibited structurally intact tissues. However, in mice exposed to AlCl_3_, the renal cortex showed signs of glomerular atrophy, cystic dilatation of the renal tubules, formation of protein tubular pattern. Additionally, the examination revealed hyaline tubular dilatation within the lumens of renal tubules, with some displaying significant distortion. Furthermore, there was a significant infiltration of inflammatory cells accompanied by severe renal histopathological damage in group H. compared to group H, mice in group FH exhibited an intact renal cortex, preserved glomerular cells, and intact renal tubules. Additionally, there was a substantial reduction in inflammatory cell infiltration, indicating a marked improvement in renal histopathological injury (Figure 5A).

### 3.6 Immunofluorescence detection of ROS in kidney tissues of mice in each group

**FIGURE 6:**
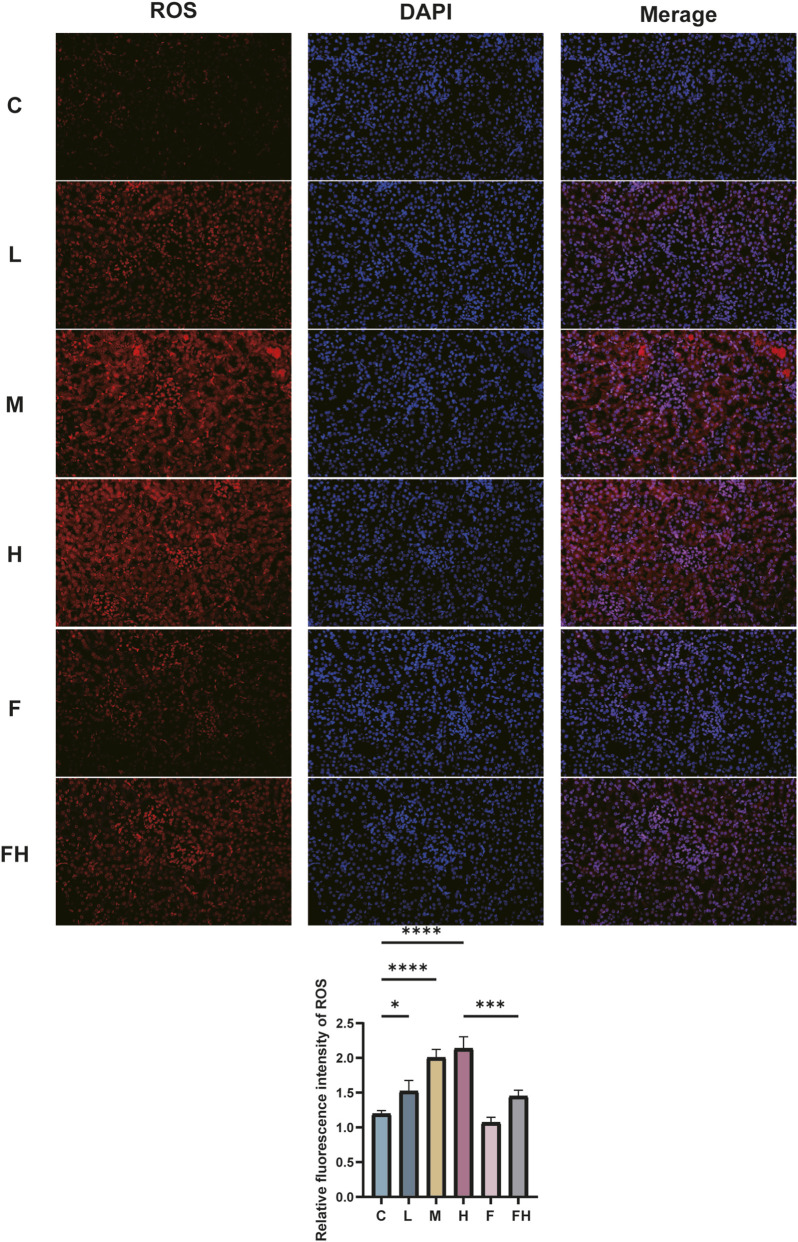
C represents the control group, L represents the low-dose AlCl_3_ exposure group, M represents the medium-dose AlCl_3_ exposure group, H represents the high-dose AlCl_3_ exposure group, F represents the fer inhibitor group, and FH represents the fer + high-dose AlCl_3_ exposure group. ROS in mice exposed to AlCl_3_ and fer intervention after AlCl_3_ exposure.

The accumulation of ROS, mainly found in the cytoplasm of renal tubular epithelial cells, is a significant characteristic of ferroptosis. Relative to the control group, the ROS expression area in the renal tissues of mice in the AlCl_3_ exposure groups progressively expanded. The fluorescence intensity elevated gradually with the increasing dose of AlCl_3_, indicating a dose-dependent effect. Compared to the control group, no significant differences were observed in ROS expression area and fluorescence intensity of renal tissues following Fer-1 treatment alone. However, the FH group exhibited varying degrees of reduction in the ROS expression area and fluorescence intensity of renal tissue compared to the H group. This implies that Fer-1 may effectively counteract ROS accumulation induced by AlCl_3_ exposure, thereby suppressing the occurrence of ferroptosis (Figure 6).

### 3.7 mRNA levels of Nrf2, GPX4, ACSL4, HO-1, FTH, and SLC7A11 in various groups of mice

**FIGURE 7:**
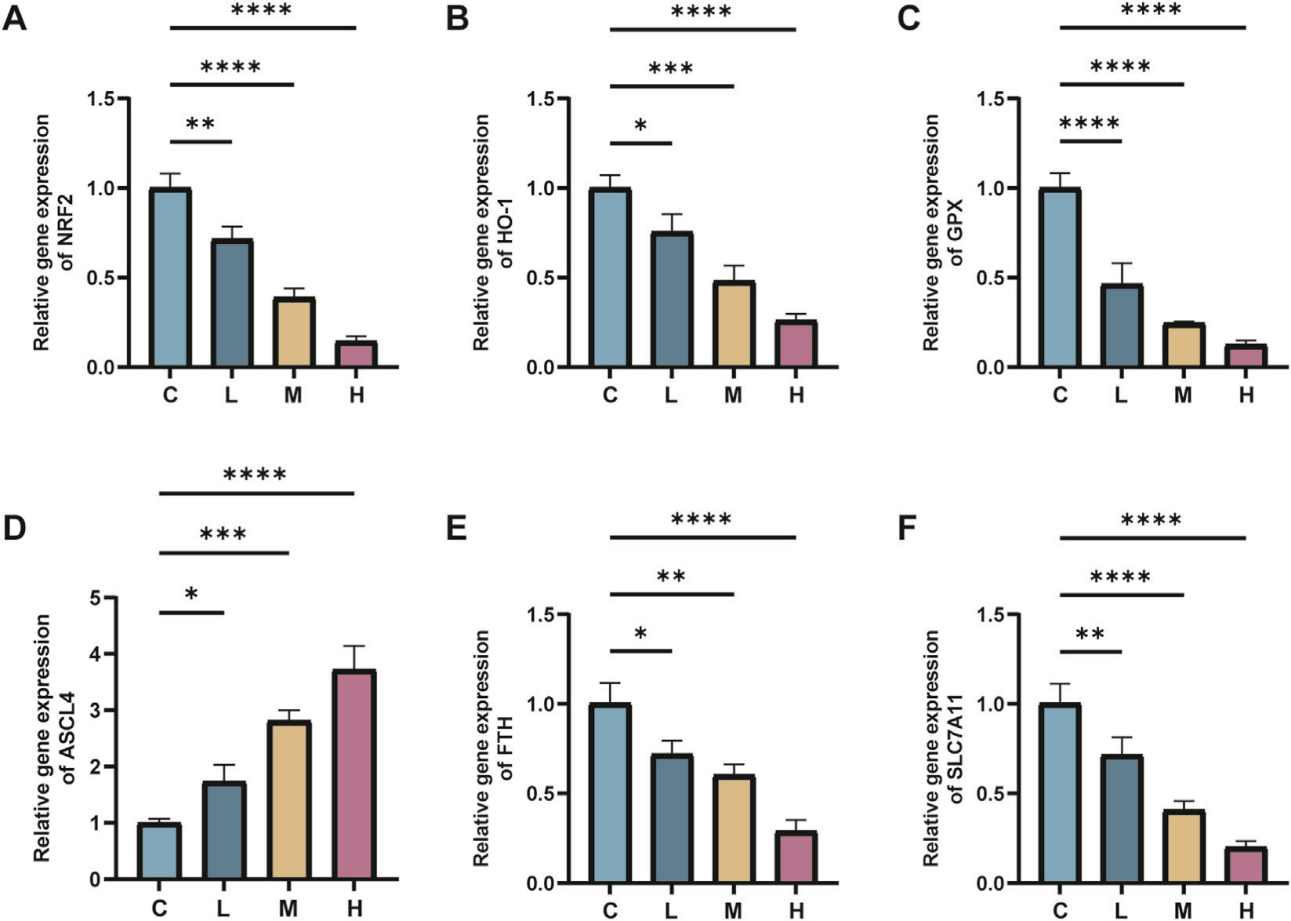
C represents the control group, L represents the low-dose AlCl_3_ exposure group, M represents the medium-dose AlCl_3_ exposure group, H represents the high-dose AlCl_3_ exposure group, F represents the fer inhibitor group, and FH represents the fer + high-dose AlCl_3_ exposure group. **(A–F)** represent the mRNA levels of Nrf2, HO-1, GPX4, ACSL4, FTH and SLC7A11 after exposure to AlCl_3_ (n = 8, *p < 0.05, **p < 0.01, ***p < 0.001, ****p < 0.0001).

**FIGURE 8:**
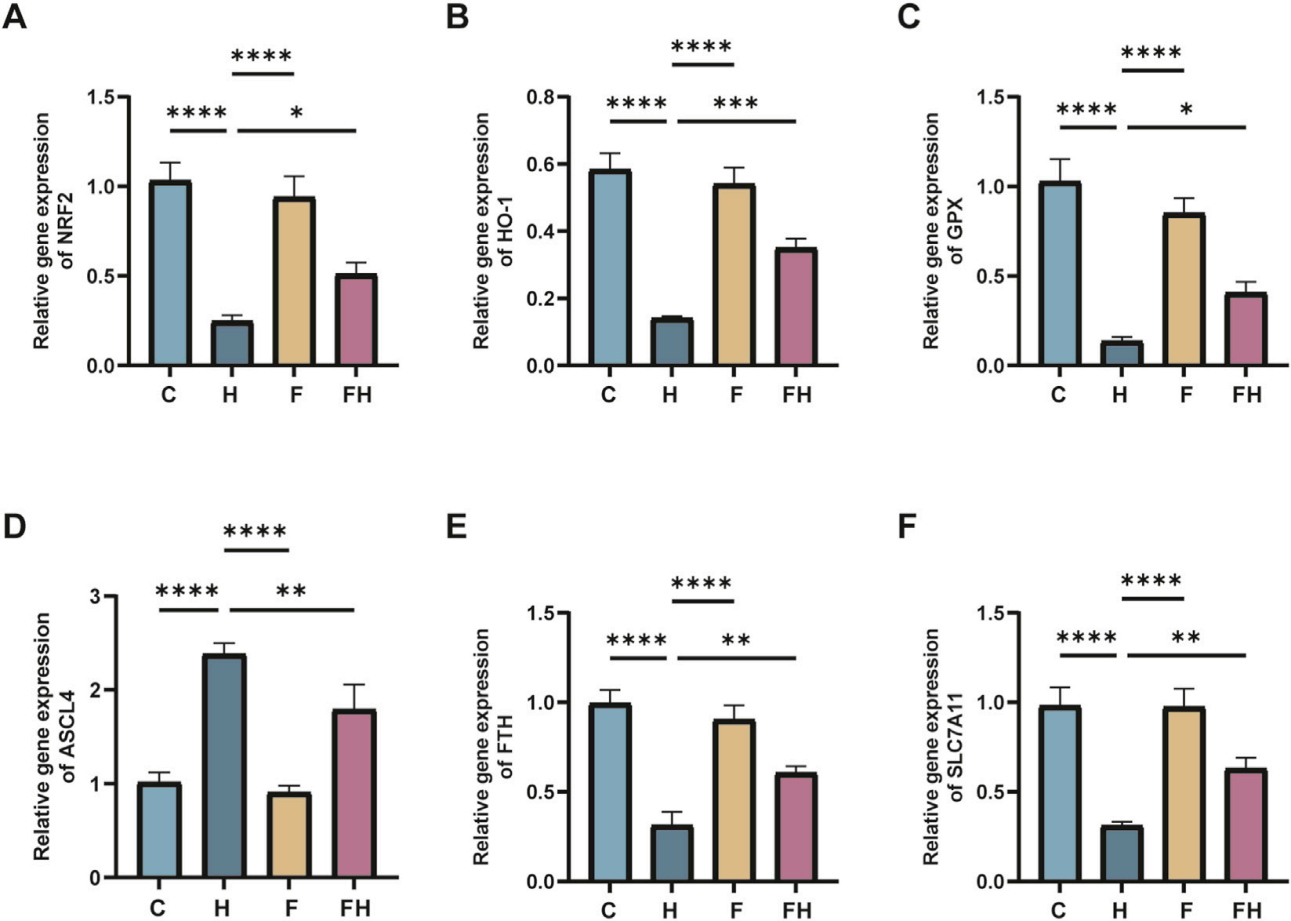
C represents the control group, L represents the low-dose AlCl_3_ exposure group, M represents the medium-dose AlCl_3_ exposure group, H represents the high-dose AlCl_3_ exposure group, F represents the fer inhibitor group, and FH represents the fer + high-dose AlCl_3_ exposure group. **(A–F)** represent the mRNA levels of Nrf2, HO-1, GPX4, ACSL4, FTH and SLC7A11 after intervention with ferroptosis inhibitors. (n = 8, *p < 0.05, **p < 0.01, ***p < 0.001, ****p < 0.0001).

The real-time fluorescence quantitative PCR findings revealed significant alterations in mRNA expression levels within renal tissues following AlCl_3_ exposure. Specifically, there was a considerable decrease in the mRNA expression levels of Nrf2, GPX4, HO-1, FTH, and SLC7A11 genes, alongside an elevation in ACSL4 gene expression, compared to the control group. These changes exhibited a dose-dependent relationship with AlCl_3_ exposure. Therefore, AlCl_3_ exposure potentially disrupts the redox response system in mouse kidney tissues by influencing amino acid, ferritin, and lipid metabolic pathways. Moreover, the mRNA expression levels of Nrf2, GPX4, ACSL4, HO-1, FTH, and SLC7A11 in renal tissues were not significantly affected by the administration of Fer-1 alone relative to the control treatment. However, compared to the H group, the renal tissue of mice in the FH group showed significantly higher levels of Nrf2, GPX4, HO-1, FTH, and SLC7A11 mRNA expression and significantly reduced levels of ACSL4 mRNA expression (Figures 7, 8). This indicates that Fer-1 intervention may alleviate ferroptosis induced by AlCl_3_ exposure to a certain extent.

### 3.8 Protein levels of Nrf2, GPX4, ACSL4, HO-1, FTH, and SLC7A11 in various groups of mice

**FIGURE 9:**
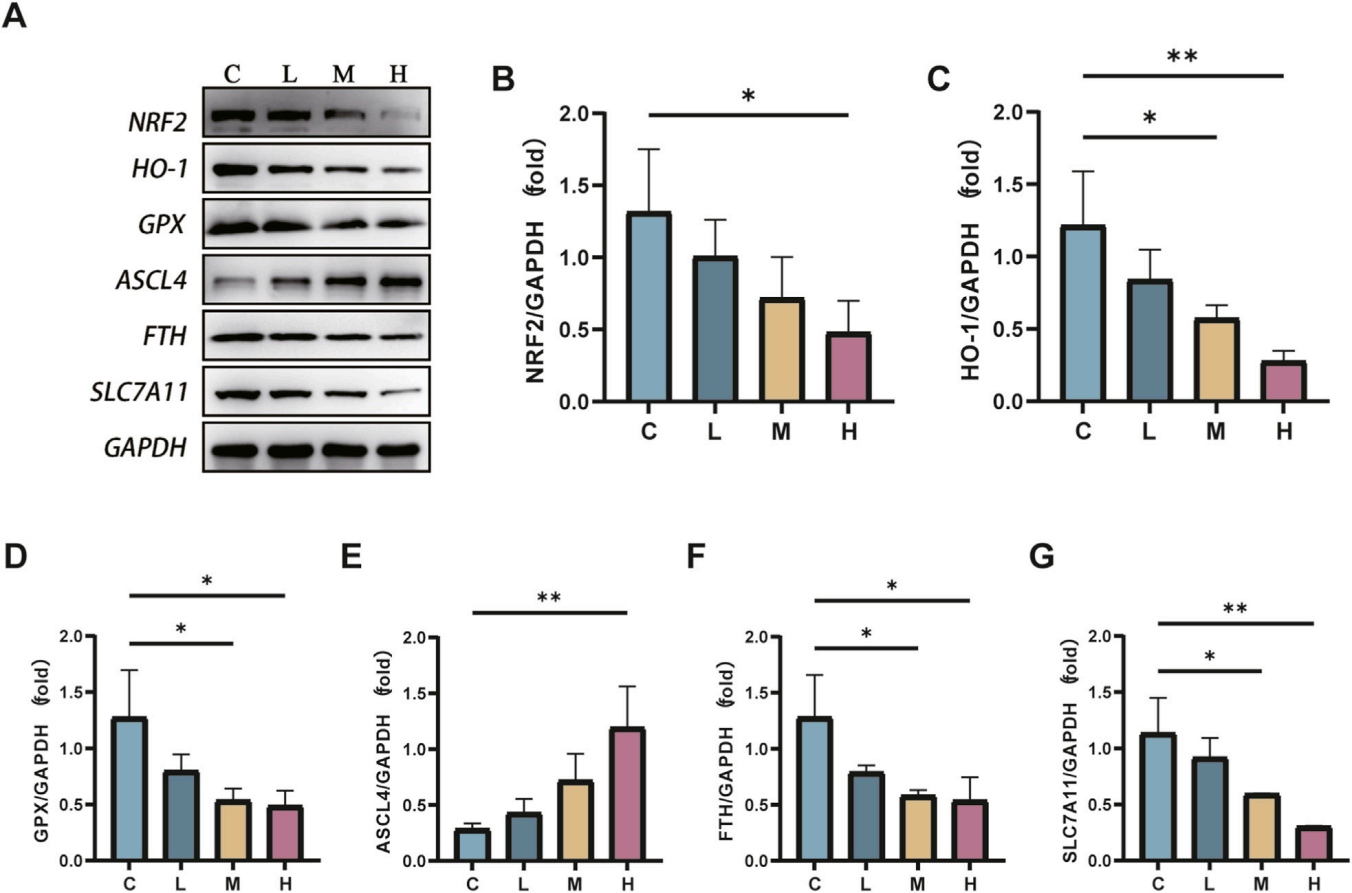
C represents the control group, L represents the low-dose AlCl_3_ exposure group, M represents the medium-dose AlCl3 exposure group, H represents the high-dose AlCl3 exposure group, F represents the fer inhibitor group, and FH represents the fer + high-dose AlCl3 exposure group. **(A)** is a protein immunoblotting graph. **(B–F)** are the quantitative expression graphs of Nrf2, HO-1, GPX4, ACSL4, HO-1, FTH, and SLC7A11 proteins after AlCl3 exposure. (n = 8, *p < 0.05, **p < 0.01, ***p < 0.001, ****p < 0.0001).

**FIGURE 10:**
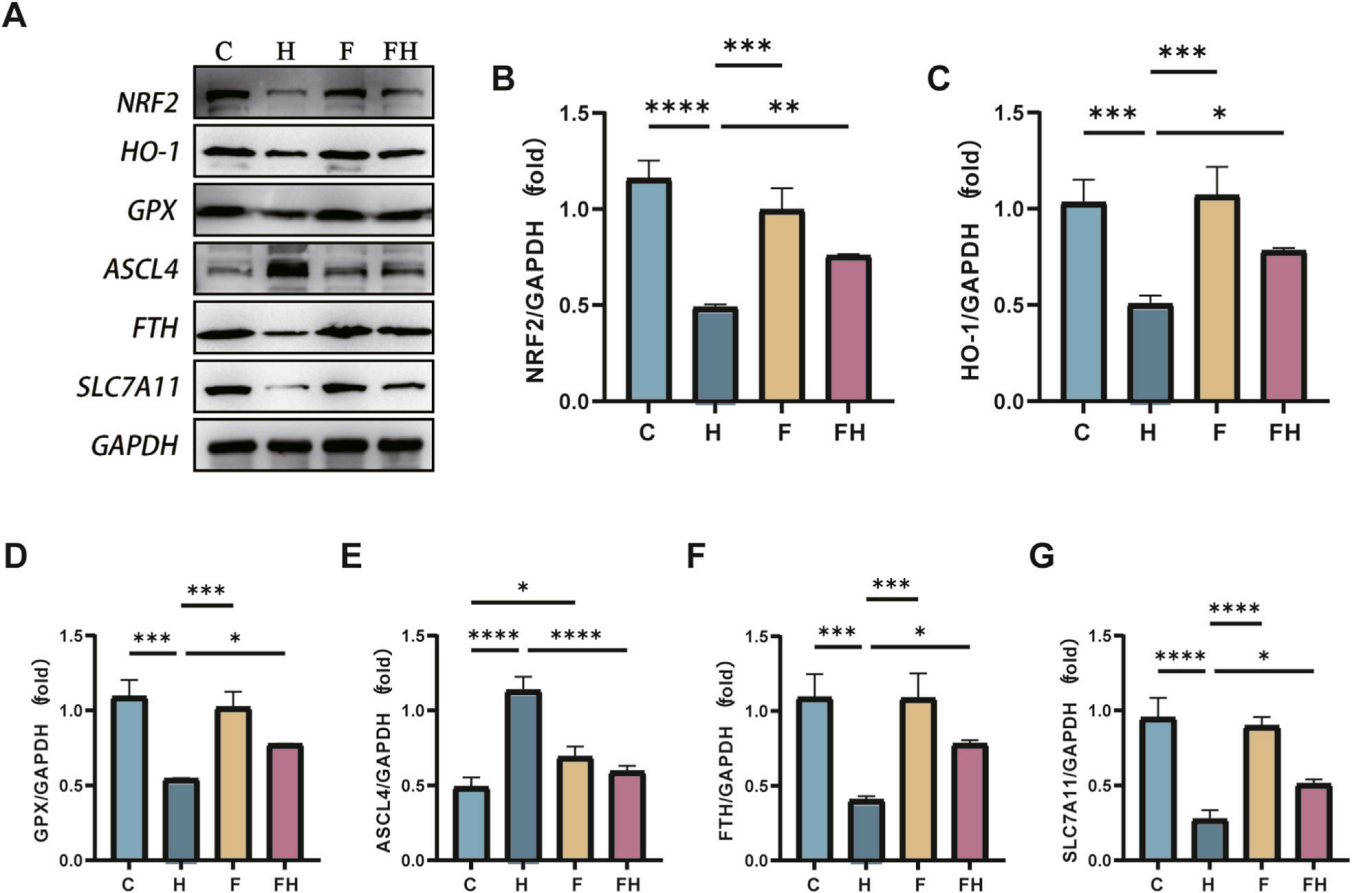
C represents the control group, L represents the low-dose AlCl_3_ exposure group, M represents the medium-dose AlCl_3_ exposure group, H represents the high-dose AlCl3 exposure group, F represents the fer inhibitor group, and FH represents the fer + high-dose AlCl3 exposure group. **(A)** is a protein immunoblotting graph. **(B-F)** are the protein quantitative expression graphs of Nrf2, HO-1, GPX4, ACSL4, HO-1, FTH and SLC7A11 after intervention with ferroptosis inhibitors. (n = 8, *p < 0.05, **p < 0.01, ***p < 0.001, ****p < 0.0001).

AlCl_3_ exposure significantly reduced the protein expression levels of Nrf2, GPX4, HO-1, FTH, and SLC7A11 in renal tissues relative to the control treatment. Conversely, it increased the protein expression levels of ACSL4 in renal tissues, exhibiting a dose-dependent effect with AlCl_3_ exposure. However, the Fer-1 treatment partially reversed this trend to some extent (Figures 9, 10).

### 3.9 Nrf2 protein expression detection in mouse kidney cells via immunofluorescence staining technique

**FIGURE 11:**
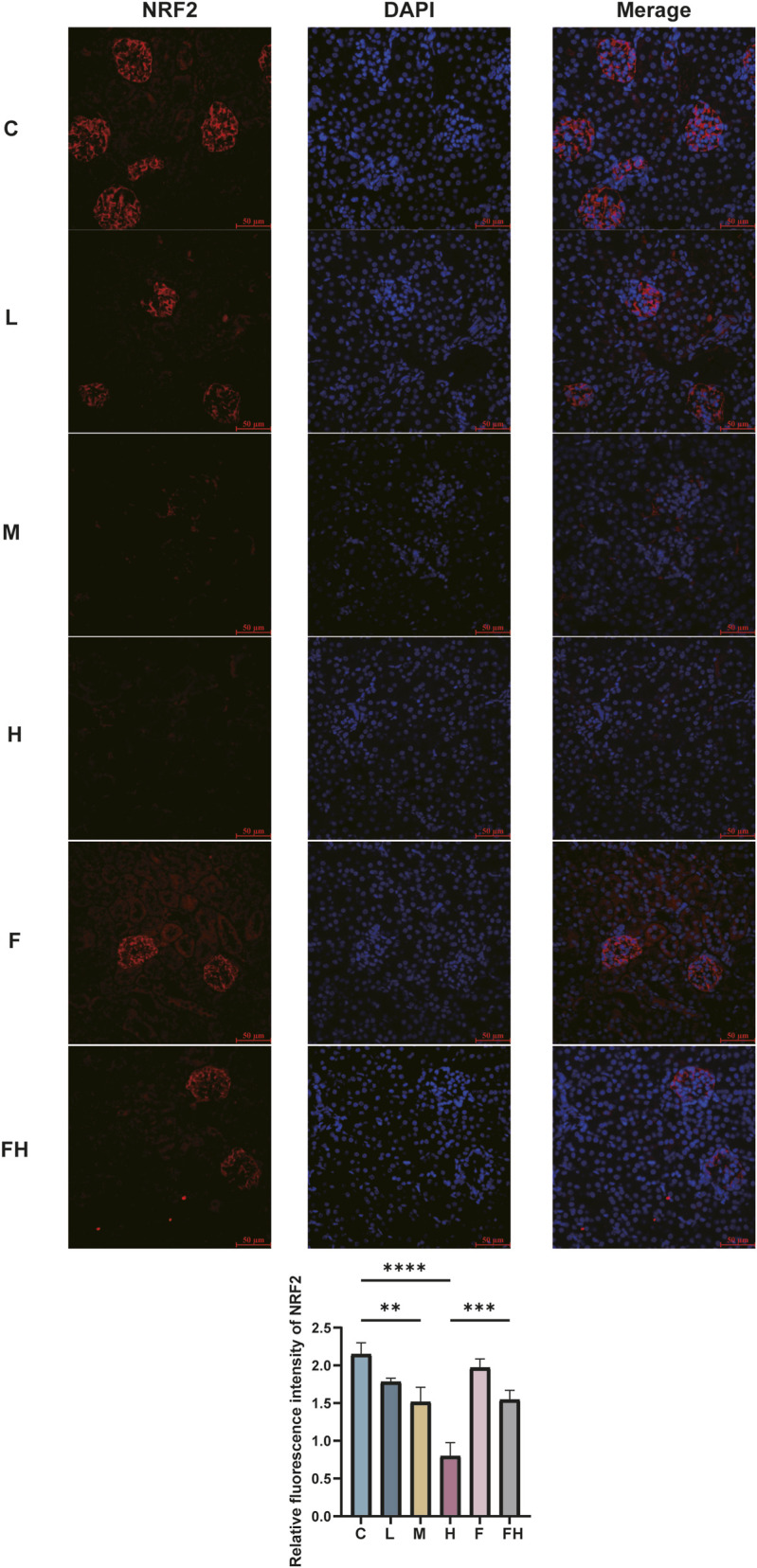
C represents the control group, L represents the low-dose AlCl_3_ exposure group, M represents the medium-dose AlCl^3^ exposure group, H represents the high-dose AlCl_3_ exposure group, F represents the fer inhibitor group, and FH represents the fer + high-dose AlCl^3^ exposure group.Intensity of NRF2 fluorescence expression in mouse kidney tissue after AlCl_3_ exposure and fer intervention.The column chart is a quantitative expression of its fluorescence intensity. (n = 8, *p < 0.05, **p < 0.01, ***p < 0.001, ****p < 0.0001).

The expression of Nrf2 in mouse kidney tissues after AlCl3 exposure and Fer-1 intervention was detected by immunofluorescence technique. In the control group, the expression of Nrf2 was high, while the expression of Nrf2 in the kidney tissues of mice exposed to AlCl3 was downregulated. However, after the intervention of Fer-1, the expression of NRF2 in the kidney tissues was restored to a certain extent, indicating that inhibiting iron death could further increase the expression of Nrf2 (Figure 11).

## 4 Discussion

Al is commonly present in consumer goods such as antacids, deodorants, food, water, and beverages. It has been utilized for centuries in various industrial sectors, including glass production, alum manufacturing, and clay processing (McDonald et al., 2021). Despite being a non-essential metallic element, excess accumulation of Al in the human body can damage several organ systems. These organs and systems include the liver, nerves, bones, kidneys, reproductive and immune systems (McDonald et al., 2021). Numerous studies have revealed that the kidneys are among the primary target organs affected by Al toxicity (Al Dera, 2016). In healthy humans, more than 95% of Al ions are eliminated by the kidneys.

During a cross-sectional study involving 77 women over 55 years of age, a positive correlation was observed between urinary Al ion levels and the levels of KIM-1, which serves as a marker of early tubular injury (Callan et al., 2015). Additionally, in an assessment of 1,434 middle-aged and older adults, plasma Al ion concentrations were significantly associated with reduced renal function. Moreover, the investigation identified a synergistic effect of simultaneous exposure to multiple metals on the impairment of renal function (Liu et al., 2020). Urinary Al concentrations among workers exposed to Al occupationally ranged from 4 to 11 μg/L. In addition, animal studies demonstrated that male rats exhibited suppressed renal function following intraperitoneal injections of AlCl_3_ (Liu et al., 2016). While numerous investigations have assessed Al-induced renal injury, the mechanism of Al-induced nephrotoxicity remains incompletely understood. However, ROS was suggested to be a crucial factor in Al-induced nephrotoxicity (Al-Kahtani and Morsy, 2019). Ferroptosis is an iron-dependent programmed cell death driven by membrane phospholipids (PL). Upregulated expression of transferrin receptor 1 (TFR-1) results in an overload of intracellular Fe^2+^, initiating the Fenton reaction. This cascade results in the excessive accumulation of ROS, reduced activity of lipid peroxidation repair enzyme GPX4, disrupted lipid peroxidation metabolism, and altered cellular composition with MDA accumulation and GSH depletion (Stockwell, 2022). Increasing evidence revealed (Wang et al., 2021) that ferroptosis might serve as an initial trigger in the progression of kidney diseases by disturbing cellular redox balance. In the present investigation, an Al-induced nephrotoxicity model was constructed utilizing mice exposed to AlCl_3_. In addition, this investigation explored the involvement of ferroptosis in Al-induced renal injury in mice. It also examined the potential mechanism of the Nrf2 pathway in the development of renal injury caused by Al-induced ferroptosis.

Our research results indicate that continuous 4 - week exposure of mice to AlCl_3_ can result in the accumulation of Al in kidney tissues. Moreover, as the concentration of AlCl_3_ exposure increases, the Al content in kidney tissues also shows an upward trend. These findings are consistent with those of Al Kahtani(Al-Kahtani and Morsy, 2019), who demonstrated that rats exposed to high doses of Al can experience Al accumulation in their liver and kidney tissues. Previous studies have shown that exposure to AlCl_3_ can lead to weight loss (Zhang et al., 2014). In this study, we also found that after AlCl_3_ exposure, the body weight and relative body weight of mice decreased in a dose - dependent manner. However, treatment with Fer - 1 can prevent the weight loss caused by AlCl_3_ exposure. The physiological structure of the kidneys is a prerequisite for normal kidney function. Meanwhile, serum creatinine and urea nitrogen are commonly used as indicators of renal function in clinical practice. Serum creatinine and urea nitrogen are metabolic wastes in the human body that are excreted through glomerular filtration. When the glomerular filtration rate decreases, serum creatinine and urea nitrogen levels increase due to their retention in the blood. Our results show that AlCl_3_ exposure can cause kidney damage, specifically manifested as changes in kidney morphology, a decreased glomerular filtration rate, and disorders of renal tubular reabsorption, which are consistent with previous studies (El-Kenawy Ael et al., 2014; Liu et al., 2016). Additionally, this study found that administration of Fer - 1 can reverse the renal function and structural damage caused by AlCl_3_ exposure, suggesting that ferroptosis may be involved in the occurrence and development of renal toxicity caused by AlCl_3_ exposure.

One characteristic of ferroptosis is the accumulation of iron. Studies have demonstrated that the use of iron chelators can reduce iron overload, thereby inhibiting induced ferroptosis (Yang et al., 2012). Additionally, ferroptosis inhibitors can ameliorate renal injury in various disease model mice by reducing tissue iron content (Dixon et al., 2012; Friedmann Angeli et al., 2014; Gao et al., 2015; Skouta et al., 2014). In this study, we detected the situation of tissue iron deposition and found that, compared with the control group, the Fe^2+^ content in the renal tissue of mice increased significantly as the dose of AlCl_3_ exposure increased, showing a dose - dependent effect. Moreover, compared with the high - dose AlCl_3_ exposure group (H group), the Fe^2+^ content in the renal tissue of mice in the high - dose AlCl_3_ exposure group supplemented with Fer (H + Fer group) was significantly reduced. This indicates that AlCl_3_ can promote iron accumulation in renal tissue, while Fer can inhibit the excessive accumulation of Fe^2+^ in renal tissue, reduce iron - mediated oxidative stress, and thus alleviate damage to renal tissue.

Heightened level of iron-dependent lipid peroxidation is another characteristic of ferroptosis. A prior investigation (Lane et al., 2018) confirmed that iron accumulation in the body exacerbates the Fenton reaction, generating significant quantities of ROS. ROS attacks lipids in membranes, increases LPO, and ultimately results in oxidative damage and cell death. Excessive production of free radicals stimulates the body to safeguard itself by producing enzymatic endogenous antioxidants (e.g., SOD and GPx) or non-enzymatic antioxidants (e.g., GSH). These antioxidants are the first line of defense against free radical-induced damage. To elucidate the impact of AlCl_3_ exposure on the lipid peroxidation level in renal tissue, we measured the levels of reactive oxygen species (ROS), malondialdehyde (MDA), glutathione (GSH), and superoxide dismutase (SOD) in renal tissue. The results revealed that in the renal tissue of mice exposed to AlCl_3_, the levels of ROS and MDA significantly increased, whereas the levels of SOD and GSH significantly decreased, exhibiting a dose - dependent effect. After intervention with Fer - 1, the levels of ROS and MDA decreased, and the levels of SOD and GSH increased.

Previous studies have demonstrated that aluminum exposure disrupts the antioxidant system, elevates ROS levels, induces lipid peroxidation, and causes kidney damage (Du et al., 2023). Additionally, aluminum intake can trigger oxidative stress, reduce the GSH content in different tissues, and inhibit the activity of antioxidant enzymes (Al-Kahtani and Morsy, 2019; Yousef et al., 2019). These findings strongly corroborate that AlCl_3_ exposure induces ferroptosis in renal tissue. Fe^2+^ and H_2_O_2_ can oxidize organic substances in the presence of ROS, generating hydroxyl radicals (HO-) through lipid peroxidation. This marks the initiation of lipid peroxidation (Ayala et al., 2014). Lipid peroxidation operates as a chain reaction propelled by free radical groups. This process produces hydroperoxides (LOOH) and regenerates a new lipid radical group, allowing the reaction to continue. Subsequently, lipid hydroperoxides are catalyzed by Fe^2+^ to produce alkoxyl groups (LO-). This disrupts the redox reaction system of the body, resulting in the accumulation of excessive lipid peroxides and inducing the onset of ferroptosis (Reis and Spickett, 2012).

Although it has been demonstrated that Al can cause ferroptosis, however, the precise mechanism remains elusive. Nrf2, a transcription factor, protects cells from oxidative damage (Mizumura et al., 2020). Under normal conditions, Nrf2 undergoes degradation via the proteasome system, primarily in a Keap1-dependent manner (McMahon et al., 2003). Elevated ROS levels induce structural alterations in Keap1, preventing it from binding to the structural domain of Nrf2 to inhibit its activity. Consequently, Nrf2 undergoes slow degradation, accumulating in the cytoplasm. As a component of the feedback loop of the antioxidant defense system, the activation of ARE (antioxidant response element) genes by Nrf2 increases. These include various genes downstream of Nrf2, such as glutathione peroxidase (GPX), solute carrier family member 7A11 (SLC7A11), and heme oxidase-1 (HO-1). These genes are commonly known to enhance the antioxidant defense system, offering protection against oxidative stress (Song and Long, 2020). In recent years, there has been a growing body of research on the relationship between Al and the Nrf2 signaling pathway. Rats exposed to Al-containing aqueous solutions exhibited oxidative damage to hippocampal tissue, alongside increased expression of Nrf2 and its downstream gene NQO1 in hippocampal cells (Dinkova-Kostova and Talalay, 2010). In SH-SY5Y cell culture, adding 100 μM Al-maltolate to the culture solution for 24 h increased the expression of Nrf2 and its downstream gene NQO1 in SH-SY5Y cells. However, both were suppressed at an Al-maltolate concentration of 200 μM (Lin et al., 2013). The above findings indicate an association between Al-induced toxicity and disrupted Nrf2 signaling. In the present investigation, decreased mRNA and protein expression levels of NRF2, HO-1, SLC7A11, and GPX4 were observed in renal tissues of AlCl_3_-exposed mice, showing a dose-dependent relationship with AlCl_3_ exposure.

Nevertheless, Fer-1 intervention increased the mRNA and protein expression levels of NRF2, HO-1, SLC7A11, and GPX4. Immunofluorescence results showed that AlCl_3_ exposure decreased NRF2 content in kidney tissue, and this trend was attenuated after Fer-1 intervention. However, this effect was mitigated after the Fer-1 intervention. Emerging evidence (Wang et al., 2021) suggests that ferroptosis, potentially serving as an early event, is pivotal in the progression of renal disease by disturbing cellular redox homeostasis and damaging renal tubular cells. The expression of NRF2 is directly associated with susceptibility to ferroptosis: upregulation of NRF2 expression inhibits ferroptosis, while its downregulation promotes it (Dodson et al., 2019; Fan et al., 2017; Sun et al., 2016). These findings imply that AlCl_3_ exposure disrupts NRF2 expression in mouse kidney tissues, thereby influencing the expression of downstream antioxidant genes and potentially triggering ferroptosis. However, Fer-1, functioning as a ferroptosis inhibitor, may mitigate the renal injury induced by ferroptosis.

Elevated levels of tissue free iron render the organism more susceptible to ferroptosis. Excessive iron in the body is stored in ferritin, comprising ferritin light chain (FTL) and ferritin heavy chain 1. Reduced FTH1 levels make cells more susceptible to ferroptosis (Yang et al., 2016). AlCl_3_ exposure can decrease mRNA and protein expression levels of FTH1 in renal tissues in a dose-dependent manner.

The study revealed that exposure to AlCl_3_ reduced the mRNA and protein expression levels of ferritin heavy chain (FTH) in renal tissues, and the extent of reduction was dose - dependent on the AlCl_3_ exposure. After the intervention with Fer - 1, both the mRNA and protein expression levels of FTH increased. When combined with the changes in tissue iron levels, it implies that aluminum (Al) may trigger ferroptosis by influencing the iron homeostasis pathway. Therefore, we hypothesize that exposure to AlCl_3_ may affect the synthesis of FTH. Moreover, the use of the ferroptosis inhibitor Fer - 1 exerted a protective effect, restoring iron homeostasis and thus inhibiting the occurrence of ferroptosis.

Lipid peroxidation is critically involved in ferroptosis, with ACSL4 being an essential gene involved in lipid metabolism. Preceding reperfusion or reoxygenation, the inhibition of ACSL4 can protect cells from harm by suppressing ferroptosis (Li et al., 2019). Furthermore, genomic investigations have underscored the significance of ACSL4 in the onset of ferroptosis (Doll et al., 2017). In the present research, both mRNA and protein levels of ACSL4 in the renal tissues of mice exposed to AlCl_3_ exhibited a dose-dependent increase. However, intervention with Fer-1 results in heightened mRNA and protein expression levels of ACSL4. It was postulated that AlCl_3_ exposure could enhance ACSL4 expression in renal tissues, thereby instigating ferroptotic injury via lipid metabolism pathways. However, NRF2 might inhibit ferroptosis by repressing lipid metabolism pathways. It was hypothesized that the NRF2 pathway may affect lipid metabolism pathways by regulating ACSL4 levels, thereby attenuating ferroptosis. Fer-1 intervention restored the expression of the NRF2 pathway as mentioned earlier, ameliorating lipid metabolic pathways and impeding ferroptosis.

Simultaneously, our research has certain limitations. For example, our study did not explore the effects of aluminum exposure on the kidneys of female mice. Moreover, no corresponding cell lines were chosen for verification at the cellular level. Additionally, the animal - level research was not comprehensive. We did not use an NRF2 activator to determine whether kidney toxicity was mainly affected via this pathway. The types of ferroptosis inhibitors used were insufficiently diverse. In subsequent research, we will attempt to select two or more inhibitors for investigation.

Regarding the research duration, our study period was relatively short. In the future research process, we will optimize our research methods and cycle. For instance, we will adopt the natural drinking approach to make the exposure method more in line with human lifestyles.

In conclusion, our study reveals a dose - dependent association between AlCl_3_ exposure and renal function impairment. AlCl_3_ exposure may inhibit the NRF2 pathway and lipid metabolism pathways, which disrupts the redox system in mouse renal tissues. This disruption leads to the accumulation of abundant lipid peroxides, triggering ferroptosis in renal tissue cells and subsequently causing a decline in renal function and pathological alterations in renal tissues. Intervention with Fer - 1 can, to some extent, mitigate the ferroptosis induced by AlCl_3_ exposure. These findings may offer a foundation for the prevention and treatment of renal toxicity stemming from AlCl_3_ - induced ferroptosis.

## 5 Conclusion

In conclusion, our findings indicate that AlCl_3_ exposure induces ferroptosis by inhibiting the NRF2 - HO - 1 pathway, thereby leading to renal toxicity. Intervention with Fer - 1 reverses this scenario, enabling a certain level of restoration of the NRF2 - HO - 1 pathway and relevant ferroptosis - related indicators. These results offer a theoretical foundation for understanding the renal toxicity caused by AlCl_3_ exposure in mice.

## Data Availability

The original contributions presented in the study are included in the article/supplementary material, further inquiries can be directed to the corresponding authors.
